# Attachment, Resilience and Life Satisfaction of University Students in Cyprus after the Fourth Wave of COVID-19

**DOI:** 10.3390/ijerph21010022

**Published:** 2023-12-22

**Authors:** Panagiotis Parpottas, Paris Vogazianos, Christos Pezirkianidis

**Affiliations:** 1 Department of Social and Behavioral Sciences, School of Humanities, Social and Education Sciences, European University of Cyprus, Nicosia 2404, Cyprus; p.vogazianos@euc.ac.cy; 2 Laboratory of Positive Psychology, Department of Psychology, Panteion University of Social and Political Sciences, 17671 Athens, Greece; christospez@hotmail.com

**Keywords:** attachment dimensions, psychological resilience, life satisfaction, university students, COVID-19

## Abstract

The COVID-19 pandemic has given rise to a large-scale crisis that has also impacted the well-being and, more specifically, the life satisfaction of university students. Factors such as attachment dimensions and psychological resilience can provide us with a better understanding of students’ life satisfaction levels during the recent pandemic. While previous literature has revealed a significant association between attachment dimensions, resilience, and life satisfaction, very few studies have attempted to address a more complex relationship among all three variables for university students, and even fewer have explored this topic during the COVID-19 pandemic. Therefore, the aim of the current study was to investigate the impact of attachment dimensions on university students’ life satisfaction after the fourth wave of COVID-19 in Cyprus, with a focus on the mediating role of psychological resilience. The sample comprised 780 university students, consisting of 323 men and 457 women, aged between 18 and 61 years. Participants were recruited electronically, and after being directed to Google Forms, they completed the ECR-R for their attachment dimensions, the RES for psychological resilience, and the SWLS for life satisfaction. The results indicated significant correlations between attachment dimensions, psychological resilience, and life satisfaction. Notably, psychological resilience was found to partially mediate the relationship between attachment anxiety, as well as attachment avoidance, and life satisfaction. Specifically, attachment anxiety and avoidance negatively affected life satisfaction, partially due to lower levels of psychological resilience. These findings are discussed in relation to the existing literature, and implications for practice are provided.

## 1. Introduction

The unprecedented crisis caused by the COVID-19 pandemic has affected all socioeconomic areas and has escalated into a humanitarian crisis that threatened millions of people worldwide. The field of higher education (HE) has not been an exception, as university students have been heavily impacted by the adverse effects of the pandemic [1].

University students’ mental health has become an increasing concern in recent years [2], and unfortunately, this concern has been exacerbated by the recent pandemic. More specifically, studies report that university students faced significant mental health challenges during COVID-19, resulting in decreases in overall well-being and particularly life satisfaction [3,4,5,6]. As a result, researchers have redirected their focus towards investigating factors that could explain the challenges to university students’ life satisfaction during the pandemic. Two such factors that could shed light on this issue are attachment [7,8] and psychological resilience [9,10].

As a concept that focuses on relationships, bonds and affect regulation, attachment theory could accurately serve in formulating a map to help us place psychological resilience closer to the heart of this theory and ultimately understand changes in university students’ LS during the pandemic. This relies on the postulation that a person’s attachment system is activated in any threat context, even if a threat is unrelated to interpersonal relationships or unmet attachment needs [11]. Internal working models of attachment (IWMs) are the mental representations of attachment relationships and affect regulation experiences and include information on a person’s beliefs, expectations, emotional reactions, and coping mechanisms. IWMs can make the response to a potential threat more predictable and are not only the basis of individual differences in attachment strategies but also act as a source of resilience. This is consistent in theory [12] and research [13]. Additionally, psychological resilience can inform how individuals evaluate the level of trust in themselves to overcome difficulties and also the belief in their ability to handle a potential or actual threat. Therefore, on a conceptual level, attachment dimensions could possibly provide us with indications of an individual’s psychological resilience, which could then explain university students’ assessments of their LF.

Although attachment dimensions were found to be significantly linked with psychological resilience [14], very few studies have attempted to investigate the impact of the two variables on university students’ life satisfaction [15]. Additionally, to the best of our knowledge, no previous studies explored a more complex association between all three variables using a mediation analysis. Moreover, there has been even less exploration of this topic during the COVID-19 pandemic. Therefore, the aim of our study was to investigate the role of attachment dimensions and psychological resilience on university students’ life satisfaction during the COVID-19 pandemic and, more specifically, after its fourth wave in Cyprus.

### 1.1. Life Satisfaction in University Students

Over the past three and a half years, the academic community has witnessed several changes in HE, particularly the psychological implications of the COVID-19 pandemic on university students’ experience. This unfortunate global reality has been well documented in several studies [16,17,18,19]. We believe that further research should now focus on better understanding the pandemic’s impact on students’ well-being.

An essential component of subjective well-being is life satisfaction (LS). LS has emerged from positive psychology as a construct that represents a cognitive evaluation of one’s life. More specifically, it highlights the individual’s subjective assessment of the satisfaction with their current life based on self-established standards [20,21]. Satisfaction from life is considered a very important parameter in HE, not only due to its positive relationship with higher academic performance but also because it has the potential to enhance students’ future expectations, overall academic experience, relationships, and psychological well-being [22,23]. However, students’ living conditions underwent a drastic transformation during the pandemic, and it is reasonable to assume that their assessment of life standards changed accordingly. In fact, studies have demonstrated that the consequences of COVID-19 were associated with negative perceptions of LS among university students [24]. Furthermore, Rogowska et al. [5] found that higher levels of anxiety during the pandemic were linked to increased stress, poorer health, and reduced LS among students. Similarly, Aslan et al. [4] reported that students experienced high levels of perceived stress, mild generalized anxiety, and low LS during the pandemic. Finally, another study revealed that during the second wave of the pandemic, university students’ LS had significantly decreased compared to the first wave [6].

### 1.2. Resilience

One factor that has captured the attention of researchers and clinicians, especially in the context of helping individuals overcome adversities, like those brought about by the COVID-19 pandemic, is resilience. This interest arises from the fact that higher levels of resilience tend to be associated with positive long-term outcomes for general health, which in turn leads to increased adaptability and LS [25].

The response to a threat indicates the levels of an individual’s resilience, and this relies on a number of factors, such as the individual’s emotional regulation skills and also their ability to flexibly adapt to distressing events. Certain personality traits have been identified to be significantly associated with resilience [26], while on a conceptional continuum, resilience is often perceived on the one end as a basic and constant personality characteristic [27], while on the other is critically interpreted as such [12,28]. Another group of scholars describes resilience as a dynamic process that stems from the interaction between individual (e.g., emotion regulation, internalization of primary caregiver figures, etc.) and external factors (e.g., family, society, environment, etc.) and results in an ability that is different from person to person [29]. While resilience is a multifaceted concept, it can be primarily understood as a response mechanism that is activated to protect individuals from stressors during intense or threatening conditions, ultimately helping them adapt to challenging experiences [30,31,32,33]. Furthermore, psychological resilience can be understood as the individual’s assessment of themselves as resilient, and two potential underlying constructs of psychological resilience are self-confidence (trust and assurance in oneself that an individual is resilient) and self-efficacy (the individual’s belief concerning their capability to cope during a stressful situation) [34]. These two specific constructs were found to be associated with positive outcomes after stressful events and were also linked with the conceptual model of resilience [34,35,36].

Research on the effects of COVID-19 on the general population’s mental health has revealed the crucial role of resilience in the reduction of psychological distress [37] and in the minimization of negative and catastrophic psychological impacts during quarantine and isolation [38]. Additionally, it was found that resilience aided in managing initial stress levels that emerged during the first wave of the pandemic [39]. Lastly, lower levels of psychological resilience were associated with depressive symptoms [40].

Similarly, studies before the pandemic emphasized the important role of resilience in HE as a protective factor, aiding students in overcoming challenges, such as adaptation difficulties, psychological distress, academic struggles, and attrition [35,41,42]. Furthermore, resilience was associated with students’ LS [15,43]. In a longitudinal study conducted several years before the pandemic, it was intriguingly revealed that resilience exhibited negative correlations with depression, anxiety, and stress, and predicted students’ positive mental health for up to one year but not beyond [44]. This suggests that while resilience played an important role in university students’ mental health, its protective capacity declined over time. Additionally, the same study demonstrated that the influence of resilience on mental health had a reciprocal effect, with both variables affecting each other.

Research during the pandemic revealed the positive impact of resilience on university students in various aspects, including stress levels, studying, interpersonal relationships, LS, and general well-being. Liu et al. [45] found that resilience, among other factors, predicted students’ well-being. Yildrim et al. [46] reported that resilience was negatively associated with depression, anxiety, and loneliness but positively linked to LS. The positive correlation between resilience and LS was further reported by a study involving university students from Pakistan [9]. Oducado et al. [47] highlighted the protective role of resilience in helping graduate Filipino students cope with the fear associated with COVID-19 and its related stress. Moreover, Quintiliani et al. [48] found that resilience skills helped students overcome pandemic-induced learning and interpersonal difficulties. Finally, another study found that during COVID-19 confinement, university students exhibited high levels of resilience regardless of their sociodemographic characteristics [49].

### 1.3. Attachment

Attachment theory postulates that the attachment system is activated in stressful situations, and the child strives for proximity with attachment figures. The activation of the attachment system leads to a primary strategy utilized by the child to search and reestablish proximity with an attachment figure (or their mental representations in the case of their physical absence). The attachment system is deactivated, or diffused, when and until security is achieved. The emotional interaction between the child and the caregiver contains information on an individual’s emotion regulation skills as well as the response strategies used to cope with emotions in any given situation [12]. It is important to note here, that Bowlby [50] assumed that this information is internalized and manifested in thoughts and behaviors. So, based on the theory, these skills and coping mechanisms can be located in cognitive representations, the so-called IWMs, which under specific conditions remain quite stable in time.

Bowlby’s theoretical propositions on relationships, bonds and affect regulation were initially studied in the interactions between infants and caregivers (attachment figures), but later applied in adult close relationships [51]. In adulthood, actual or symbolic threats, on a conscious or an unconscious level, can activate the attachment system and IWMs can inform emotional reactions and affect regulation and proximity behaviors. Depending on their IWMs, adults can develop a sense of attachment security or insecurity. Individual differences in these reactions can be understood in two attachment dimensions: attachment anxiety and attachment avoidance. Attachment anxiety is associated with the fear of rejection and an excessive need for approval and is characterized by hyperactivating attachment strategies. These are compensatory strategies used to regulate emotion by excessively depending on others to obtain support. On the contrary, attachment avoidance is associated with deactivating attachment strategies, which include an excessive need for self-reliance, fear of depending on others, and the downregulation of emotions in an effort to deny any attachment needs.

According to theoretical postulations, attachment can be seen as a core feature of resilience [50]. More specifically, the physical and emotional availability of a person who acts as a source of security in childhood, and later access to mental representations of this interaction, results in a sense of felt security. Feeling internally secure leads to successful affect regulation, which helps individuals maintain emotional balance and personal adjustment in the face of stressful events and such experiences act as a source of resilience. On the other hand, attachment insecurity is considered as a risk factor that reduces resilience at times of stress. Thus, a possible connection of attachment characteristics with psychological resilience relies on the fact that the information consisted in IWMs (i.e., emotional regulation, self-esteem, closeness in relationships, support, felt security, etc.) render attachment as one of those individual attributes that can foster resilience [14].

### 1.4. Attachment, Resilience, and Life Satisfaction

Previous studies with young adults reveal a connection between psychological resilience and attachment organization (in some studies, it can be found as attachment styles, patterns, or dimensions) as well as attachment and LS. Specifically, Yu et al. [52] found that the parent–child attachment was positively correlated with college students’ psychological resilience. Similarly, Shibue and Kasai [53] found a significant correlation between attachment styles and psychological resilience, with the subscales of resilience having a strong and positive correlation with the secure style and a negative correlation with the two insecure styles of attachment. Also, another study revealed that secure attachment predicted psychophysiological resilience [54]. Furthermore, a number of studies that show the existence of a statistically significant relationship between attachment and psychological resilience can be found in the systematic review of Darling-Rasmussen et al. [14].

Besides the connection between attachment and resilience, research shows that attachment styles have also been linked with LS. More specifically, past research suggested that attachment anxiety and avoidance were negative predictors of university students’ LS [15]. Even though attachment security is considered as a source for developing resilience to deal with challenges, and as both variables can predict LS, to the best of our knowledge there is only a scant number of studies addressing a more complex association between all three variables. More importantly, we have not found any such studies in the field of HE, before or during the COVID-19 pandemic. Nevertheless, some of the existing studies provide us with some indications of the association between the three variables. For example, the study of Lane [55] found that among other variables, attachment anxiety and avoidance, as well as ego resilience, were significant predictors of LS and other outcome variables in a sample of college students. Additionally, Tu et al. [56] found that the relationship between loneliness and LS during COVID-19 differed significantly between Thai and Chinese college students because of the mediating effect of resilience, while Tamarit et al. [57] found that resilience and LS had a mediating role in the relationship between COVID-19 worries and emotional symptoms in a sample of adolescents and young adults (12–25 years old). Finally, the only study that found that psychological resilience was a mediator in the relationship between attachment styles, LS, and psychological distress was published in 2016 but in a sample of high-school students [58].

### 1.5. Aim and Research Hypotheses of the Current Study

Based on the previous literature, the current study aimed to investigate the role of attachment dimensions and psychological resilience on university students’ LS in Cyprus during COVID-19. The protective aspect of attachment security and also the potential growth and development of psychological resilience transfuse important clinical implications. It is important to note that although our study took place during the pandemic, which has been rightfully characterized as a highly global threat and a distressing condition [59,60], our data were collected after the fourth wave. Although university students had already experienced intense and harsh restrictions during the initial strikes of the pandemic, the period of our data collection still posed a number of threats for students in Cyprus, such as prolonged social distancing measures, an uncertain timeframe for resolution, financial, and academic instability. Therefore, these unique conditions provided the opportunity to understand the students’ existing appraisals of LS under the impact of attachment dimensions while exploring the role of psychological resilience in this relationship. Our research hypotheses were the following:

1. Attachment dimensions (anxiety and avoidance) will be associated with psychological resilience and LS.
2. The relationship between attachment anxiety and LS will be mediated by psychological resilience.
3. The relationship between attachment avoidance and LS will be mediated by psychological resilience.

## 2. Materials and Methods

### 2.1. Participants

The sociodemographic characteristics of the sample were collected with questions concerning gender, age, nationality, family status, residence status, level of study, program of study, and year of study. The sample comprised 323 (41.4%) men and 457 (58.6%) women, aged from 18 to 61 years (median = 21, IQR = 3.00). Participants’ nationalities were 357 (45.8%) Cypriots and 423 (54.2%) Greeks. The vast majority, 688 (88.2%), were single, with 80 (10.3%) being married/engaged, 10 (1.3%) divorced, and 2 (0.3%) widowed. Of most participants, 369 (47.3%) lived alone, with 108 (13.8%) living with a partner, 216 (27.7%) living with their parents, and 87 (11.2%) living with a roommate. The academic level of studies of the participants was 49 (6.3%) at the diploma level, 611 (78.3%) at the bachelor’s level, 114 (14.6%) at the master’s level, and only 6 (0.8%) at the Ph.D. level.

### 2.2. Measures

Experiences in Close Relationships-Revised (ECR-R). ECR-R was utilized to assess adult attachment [61] and, more specifically, the Greek version (G-ECR-R) of the questionnaire [62]. ECR-R has 36 items, and participants state how much they agree or disagree with each item on a 7-item Likert scale (1 = “Strongly disagree” and 7 = “Strongly agree”). This self-report questionnaire is designed to assess differences in attachment, providing scores for two dimensions: anxiety (how much people feel secure or insecure according to the frequency of their close people’s availability and responsiveness) and avoidance (how much people feel comfortable or uneasy in closeness to their close ones). Sample items for attachment anxiety include “I worry that others won’t care about me as much as I care about them” and, for attachment avoidance, “I prefer not to show others how I feel deep down”. ECR-R originally measures adult romantic relationships, but it is possible to adjust questions according to the study’s aim, e.g., interpersonal, parental, etc. [61]. Therefore, in the current study, participants were asked to complete each item, having in mind their close others, such as close friends, parents, siblings, relatives, and partners. G-ECR-R had good internal consistency with Cronbach’s alpha = 0.91 for both attachment avoidance and attachment anxiety [62].

Resilience Evaluation Scale (RES). Psychological resilience was assessed by the RES [34], which was translated into Greek [63]. The RES has nine items, which are completed on a 5-point Likert scale (0 = disagree, 5 = completely agree), with higher scores indicating greater psychological resilience. Scores are also provided for two subscales: self-confidence and self-efficacy. Self-confidence refers to a general feeling of certainty and trust in being resilient (sample items: “I have confidence in myself” and “I believe in myself”). Self-efficacy refers to the individual’s positive beliefs concerning their capability to adaptively cope with stressful situations (sample items: “After setbacks, I can easily pick up where I left off” and “I can cope well with unexpected problems”). The RES had a high internal consistency with a = 0.898 (self-confidence a = 0.89 and self-efficacy a = 0.87) and a very good convergent validity with other scales, such as resilience (RS), rs = 0.74; self-efficacy (GSE), rs = 0.73; self-esteem (RSES), rs = 0.71; global functioning (GF), rs = 0.55; and PTSD symptoms (PCL-5), rs = –0.39.

Satisfaction with Life Scale (SWLS). To measure LS, we used the SWLS [20]. The SWLS consists of five statements and assesses the level of LS, allowing each individual to provide a subjective report of their LS at the given time of measurement. Sample items include “In most ways my life is close to my ideal” and “I am satisfied with my life”. The items are answered on a 7-point Likert scale (1 = Strongly disagree, 7 = Strongly agree), with higher scores indicating greater LS. According to Diener et al. [20], the SWLS has high internal consistency with a = 0.87 and very good convergent validity with other scales measuring subjective well-being with r = 0.50 to r = 0.75. The scale has been translated into Greek and is available from Ed Diener’s website. The psychometric properties of the Greek scale are considered satisfactory with Cronbach a = 0.84 and a very good convergent validity with the scale of LS -LSI with r = 0.77 [64].

### 2.3. Design and Procedure

This study used a non-experimental, cross-sectional design. The first research hypothesis tested the association between the variables of attachment dimensions (anxiety and avoidance), psychological resilience (self-efficacy, self-confidence, and total resilience), and LS. Finally, to test the other two research hypotheses, we conducted a mediation analysis to see whether the effect of the independent variables (attachment dimensions) on the outcome (LS) could be mediated by resilience.

The study received ethics clearance and approval by the National Bioethics Committee of Cyprus. The committee deemed that its procedures were in accordance with requirements for research involving human participants.

Participants were recruited electronically with posted announcements via social media (e.g., Facebook, Instagram). Interested individuals were directed to Google Forms, where they could find information about the purpose of the study. Those who decided to participate, prior to completing the internet-based questionnaire, provided their signed consent and were assured that their participation would be anonymous. Data were collected between 31 March 2022 and 31 July 2023.

After their collection, all electronic data were downloaded from the platform, and subsequently, the platform was deleted. Afterwards, the electronic data were coded on an SPSS file and secured on a password-protected USB that was only accessible to the researchers.

### 2.4. Statistical Analyses

For the statistical analysis, the statistical package IBM SPSS version 29 was used (IBM, Armonk, NY, USA). Before testing our study’s hypotheses, we performed normality tests using the Kolmogorov–Smirnov test, and we also used histograms, Q–Q plots, and skewness and kurtosis. Our results indicated that data were not normally distributed, with all *p*-values being <0.001. Secondly, we tested the internal consistency of our questionnaires (G-ECR-R, RES, and SWLS) using Cronbach’s alpha, and we ran preliminary analyses for the descriptive statistics of the study. Finally, to test our hypotheses we ran a non-parametric correlation (Spearman rho) and concerning mediation analyses, we used the PROCESS plugin and followed the procedure as described in Preacher and Hayes [65].

## 3. Results

### 3.1. Reliability Analysis

All questionnaires used in this research showed excellent reliability, given the sample size as well as the number of questions that each dimension utilized, with internal consistency as measured by Cronbach’s alpha being α = 0.916 for attachment avoidance, α = 0.906 for attachment anxiety, α = 0.907 for resilience total, α = 0.872 for resilience self-efficacy, α = 0.850 for resilience self-confidence, and α = 0.899 for life satisfaction. The internal consistency values of the questionnaires are presented in Table S1.

### 3.2. Descriptive Statistics

The descriptive statistics for our sample’s demographic characteristics, including the means, standard deviations, and other characteristics of our study’s variables, can be found in Tables S2 and S3.

### 3.3. Inferential Statistics

#### 3.3.1. Correlations

Spearman’s rank correlation was computed to assess the relationship between all total scores of the questionnaires. A negative correlation was found between attachment anxiety and the variables of life satisfaction, ρ(778) = −0.371, *p* < 0.001; resilience self-efficacy, ρ(778) = −0.264, *p* < 0.001; resilience self-confidence ρ(778) = −0.377, *p* < 0.001; and total resilience, ρ(778) = −0.335, *p* < 0.001. Similarly, a negative correlation was found between attachment avoidance and the variables of life satisfaction, ρ(778) = −0.440, *p* < 0.001; resilience self-efficacy, ρ(778) = −0.289, *p* < 0.001; resilience self-confidence, ρ(778) = −0.360, *p* < 0.001; and total resilience, ρ(778) = −0.341, *p* < 0.001. Finally, a positive correlation was found between life satisfaction and all subscales of resilience, such as self-efficacy, ρ(778) = 0.485, *p* < 0.001; resilience self-confidence, ρ(778) = 0.591, *p* < 0.001; and total resilience, ρ(778) = 0.563, *p* < 0.001. All correlations can be found in Table S4.

#### 3.3.2. Mediation Analyses

The results presented in Table S5 show that there was a significant total effect of *R*^2^ = 0.133 between attachment anxiety and life satisfaction (B = −2.2738, *p* < 0.001) through the indirect path attachment anxiety to resilience total to life satisfaction ((B = −1.0001, *p* < 0.001) with (a) attachment anxiety on resilience total (B = −1.9863, *p* < 0.001) and (b) resilience total on life satisfaction (B = 0.5035, *p* < 0.001) being both significant, and the direct effect attachment anxiety to life satisfaction (B = −1.2737, *p* < 0.001). In addition, the Sobel test for the indirect effect is z = 8.054, *p* < 0.001. The mediation analysis is depicted in Figure S1.

Additionally, the results in Table S6 show that there was a significant total effect of *R*^2^ = 0.191 between attachment avoidance and life satisfaction (B = −2.7272, *p* < 0.001) through the indirect effect attachment avoidance to resilience total to life satisfaction (B = −1.0316, *p* < 0.001) with (a) attachment avoidance on resilience total (B = −2.1668, *p* < 0.001) and (b) resilience total on life satisfaction (B = 0.4761, *p* < 0.001) being both significant, and the direct effect attachment avoidance to life satisfaction (B = −1.6956, *p* < 0.001). In addition, the Sobel test for the indirect effect is z = 8.571, *p* < 0.001. The mediation analysis is depicted in Figure S2.

## 4. Discussion

The psychological impact of the crisis caused by COVID-19 has been a major concern in HE since university students were among those populations that were heavily affected. Therefore, a recent line of research is now exploring potential protective and risk factors that could have a catalytic effect on stressors created by the pandemic and which have affected students’ well-being. Our research attempted to investigate the role of attachment dimensions and psychological resilience on university students’ LS, and to the best of our knowledge, this is the first study to examine such a relationship using mediation analysis. Consistent with previous findings, our findings showed that both attachment dimensions (anxiety and avoidance) significantly and negatively correlated with psychological resilience and LS. In addition, psychological resilience and its components (self-confidence and self-efficacy) positively correlated with LS. Most importantly, it was revealed that psychological resilience partly mediated the relationship between attachment dimensions and LS.

Our study took place after the fourth wave of the pandemic, and although students had already experienced the abrupt phases of the pandemic before our study (i.e., repeated lockdowns, restrictive measures for social distancing, and reduced physical interaction with others, etc.), a number of restrictions and also the general threat of the pandemic were still active. Challenges such as keeping distance from classmates, wearing masks, the cancellation of classes due to confirmed or suspected cases of COVID-19, the fear of the virus, and generally the instability caused in the academic environment with the continuation as well as the unpredictable course of the pandemic, were among those we observed in Cyprus at the time. Exactly during this period, students high in attachment anxiety and avoidance reported that their satisfaction from life was not ideal.

### 4.1. Correlation of Attachment Dimensions, Resilience, and LS

Beginning with our first hypothesis, as expected, we found a significant correlation between all three variables. Consistent with previous studies, we found a negative correlation between attachment dimensions, resilience, and LS [12,49,50,51,52] and a positive correlation between resilience and LS [9,12,34,37]. Our results indicate that university students who have higher attachment avoidance and anxiety tend to report lower levels of psychological resilience and LS, while students with higher levels of psychological resilience tend to report higher levels of LS. Having an insecure attachment seems to relate to students’ negative beliefs in trusting themselves and their ability to respond effectively to adversities in order to adjust and enjoy life.

### 4.2. Psychological Resilience as a Mediator in the Relationship between Attachment Dimensions and LS

Our last two hypotheses were also confirmed, with our findings suggesting that psychological resilience was a significant partial mediator in the relationship between attachment insecurity and LS. Specifically, our finding confirmed the indirect effect of attachment anxiety on LS through psychological resilience, and similarly, psychological resilience mediated the effect of attachment avoidance on LS. Therefore, students who reported higher levels of attachment anxiety and avoidance showed lower levels of LF after the fourth wave of the COVID-19 pandemic, and this was partly explained because of students’ lower psychological resilience and its components of self-confidence and self-efficacy. Our findings are consistent with theoretical assumptions [64] that insecure attachment could reduce resilience and have a negative impact on well-being, which is directly linked to life satisfaction. Although this may apply to students with higher attachment insecurity, our findings can be interpreted on the basis of differences for each attachment dimension.

Individuals with higher attachment anxiety tend to intensify their emotional reactions and present an overdependence on others for protection due to their characteristic hyperactivating strategies [66]. Previous research has shown that individuals with higher attachment anxiety perceive stressful events as more threatening and tend to use less effective coping strategies [11] but also their attachment-anxiety-related strategies provoke cognitive responses that heighten negative affect and keep their attention focused on threats [67,68]. It is possible that students higher in attachment anxiety may have struggled at the time, and based on their attachment-related anxiety, they may have not been able to regulate their emotions. Additionally, according to the characteristic patterns of their attachment dimensions, their response may have been to over-rely on people who were either physically around or in remote communication (e.g., parents, partners, roommates, peers, and instructors), rather than finding more adaptive strategies to cope independently with difficulties. Consequently, their attachment anxiety could have guided their emotional and behavioral reactions and negatively affected their psychological resilience (self-confidence and self-efficacy), resulting in perceptions of not being fully confident and positive that they had the necessary recourses to remain resilient. The effect of their attachment on their psychological resilience can be explained by the negative model of self and positive model of others [69], which may have played an important role in perceiving that the situation exceeded their coping abilities and resources, in the sense of being self-doubtful and needing to dependent on others for protection. Complementary to the previous view might be the self-determination theory [70]. Based on this theory, it can be suggested that individuals high in attachment anxiety lacked previous securely stable and reliably supportive experiences, which could foster competence, healthy relatedness, autonomous behavior, and emotional self-regulation, which could then lead to strong resilience. Therefore, their difficulty in believing in themselves and relying on their existing capabilities to adaptively respond to threats reflected lower levels of psychological resilience, which eventually may have resulted, partly at least, in the negative appraisal of their life. This may explain why they reported that the standards they subjectively established for their lives were not fully met and hence why they were less satisfied with their life during that period.

Individuals with higher attachment avoidance tend to deflect attention from emotions, suppress negative feelings, and utilize compulsive self-reliance due to their characteristic deactivating strategies [66]. Therefore, students higher in attachment avoidance may have minimized the challenges experienced at the time by suppressing their emotions. Additionally, social distancing and preference for isolation may have acted as a buffer, which may have served them to minimize the discomfort of closeness. However, this avoidant stance could have created potential strains in their personal relationships and in their academic interactions and obligations. At the same time, getting in touch with the realization of their inability to fully manage their emotions, could have led to feelings of further discomfort. Hence, in the long term, maintaining their characteristic attachment pattern of distancing and emotional suppression could negatively affect their psychological resilience and this was confirmed by our results. Specifically, students higher in attachment avoidance showed that they were not able to securely rely on themselves (self-confidence of resilience) or have positive perceptions that they had the proper adaptive mechanisms to manage difficulties (self-efficacy of resilience). Paradoxically, although our findings can be interpreted by the negative model of others, they cannot be fully explained by the positive model of self [67,68] unless the positive self-model is understood as the result of defensive self-enhancement [66]. This defensive aspect can be seen in the concept of vulnerable narcissism, where a positive self-image is developed in response to close others’ unaffectionate stance and the individual’s subsequent fear of rejection. Narcissistic vulnerability is managed by avoiding closeness and intimacy, and because the ability to withstand and tolerate difficulties relies on temporary avoidant defenses that fail, it leaves the self-structure still vulnerable [71]. Similarly, according to self-determination theory [70], it can be suggested that previous unresponsive and less empathetic experiences with close others could not foster true competence to avoidant individuals, but instead, it could result in a rather self-inflated ego, in a distancing relatedness, in a compulsive unhealthy autonomous behavior, and in a suppressing emotional regulation, which ultimately may have led to lower levels of resilience. Being less adept at perceiving themselves as truly psychologically resilient can partly explain why students with higher attachment avoidance rated their lives as less satisfactory at the time.

As we have seen from our results, insecure attachment dimensions impacted students’ psychological resilience, which then negatively affected their LS. It seems that insecure attachment can be considered as a risk factor that reduces healthy responses to threats and this creates a chain effect in reducing resilience levels, which then, partly at least, negatively affects the perceived satisfaction with life. This indicates that students in our study may have searched for personal resources to overcome the adversities of the time, but insecure attachment turned out to be a risk factor for some of them, which may have affected important aspects of their lives. Finally, the partial mediation of resilience on attachment and LS may mean that other factors can affect this relationship too and that psychological resilience cannot fully explain this effect.

## 5. Limitations

The findings of the current study cannot be understood without being placed in the context of several limitations. Firstly, the data were collected from Cyprus, and generalizations cannot be applied to different cultures or to all university students. Additionally, questionnaires were completed only in Greek, and thus only Greek-speaking students could participate. As Cyprus is now a very attractive university destination, an important number of international students come to study in Cyprus. Unfortunately, international students were excluded, and this omission prohibited us from understanding the concepts of this study for students who studied abroad during the pandemic. Another limitation was that although data were collected between 2022 and 2023, lockdown conditions and other restrictive measures were not as strict in Cyprus as they were back in 2020 or in 2021. For example, the mandatory use of masks indoors, testing and presenting a safe pass, restrictions on the number of people gathering in one place, and testing and isolation measures for positive cases and close contacts were some measures enforced during the first half of 2022, whereas in the second half of 2022, these measures were gradually lifted and fully abolished until May 2023. Therefore, one could argue that the phase of data collection was a transitional period. Perhaps measurements at different times during the pandemic (e.g., after the first lockdown, and pre- and post-time of each subsequent lockdown) and also a detailed investigation of the type of threat posed by each stage of the pandemic could have provided us a better indication of the pandemic’s impact on our results. Additionally, a different methodological design could have provided us with a possible representation of the activation of the attachment system and resilience and its influence on university students’ LS during the pandemic. Fourthly, the findings were derived from online self-report questionnaires, which are based on subjective perceptions. Moreover, although the questionnaire utilized for resilience had good reliability and was easy and fast to use, it is very new, and more studies are needed for its establishment. Most previous studies used other resilience scales, e.g., CD-RISK [72], and perhaps our findings may not be fully comparable to other studies. Nevertheless, we believe that the RES scale used in our study is a valuable instrument for research and clinical purposes. A sixth limitation might be the cross-sectional design of our study, which prevents us from drawing definite conclusions concerning causal relationships. Finally, another limitation might have been the omitted data that could have influenced our findings, such as the identification of students with disabilities, studying in part-time or full-time mode, in distance or conventional method, and also the identification of using any support aids during the pandemic such as counseling, therapy, or mentoring.

## 6. Suggestions for Further Research

Based on our findings, a number of recommendations for future research could be provided. Firstly, future research needs to address issues concerning the aftermath of the pandemic in students’ LF and university adaptation. Secondly, researchers in countries that attract international university students are encouraged to explore and understand research concepts related to our topic, as important conclusions could provide implications for their academic life and general well-being. Additionally, more exploratory studies could be developed to empirically investigate how university counseling centers could facilitate the development of students’ resilience skills based on their attachment dimensions to help them increase their life satisfaction and generally their academic experience in HE. Finally, future studies may wish to use other research methodologies (i.e., qualitative, experimental) to approach similar topics and also address other possible mediation and moderator variables to explore complex relationships between attachment and LS.

## 7. Conclusions and Implications for Practice

In conclusion, our study revealed that attachment dimensions, both anxiety and avoidance, had an important effect on university students’ LF, and that this effect was partially mediated by psychological resilience. Our results highlight the importance of understanding attachment insecurity and low levels of psychological resilience as a risk factor for students’ LS. Thus, universities and other professionals should carefully assess their students’ attachment dimensions, and extra attention should be given to the provision of supportive means to develop and strengthen psychological resilience.

In light of our study’s findings, some practical implications could be suggested to inform HE institutions as well as the practice of professionals who work with university students. Our study confirmed past studies by indicating that insecurely attached students may be considered at risk as they may present lower levels of psychological resilience and negative appraisals of LS. This could ultimately affect their university adjustment and academic experience [73,74]. As university students face several challenges in their transition to HE and also during their studies, we believe that promoting a more holistic approach to facilitating personal growth might be an important area that universities need to work on. Past studies found a link between personal growth with LS, well-being, and adjustment, among other factors [75,76]. Therefore, universities must develop a compatible “mindset” of values, which reflects Maslow’s pyramid of higher needs (i.e., promote self-actualization), in order to create the necessary conditions to identify their students’ needs and facilitate personal development. Moreover, as universities are highly research-driven, they could proceed with more empirical studies to understand possible risk factors, especially during challenging times. Thus, research can inform universities’ support mechanisms to tackle risk factors and also promote personal development and growth, which could ultimately improve university students’ LS, resilience, and, generally, mental health and well-being.

Our study also has the potential to inform professionals who work therapeutically with university students. Generally, attachment theory and research can serve as a solid foundation for understanding the underlying factors that contribute to the development of emotional difficulties and can also inform clinical work [68]. Previous research suggests that psychological therapy provided in university counseling settings is very useful especially because students’ psychological distress is often associated with insecure attachment [77]. Therefore, based on our results, university counseling centers, and other professionals who offer off-campus counseling services, can help students with high attachment anxiety and avoidance understand how previous experiences shaped their responses to thread and their coping strategies in alleviating distress. Being able to make the necessary connections and identify their characteristic attachment patterns and strategies, students can develop a healthier affect regulation and also receive a corrective emotional experience that will meet their basic psychological needs. For example, students higher in attachment anxiety can recognize their hyperactivating strategies and work with their fears of helplessness and hopelessness. Additionally, therapy can help them break the insecure pattern of managing their emotions and develop a more autonomous stance by focusing on self-efficacy and self-confidence, with less overreliance on others. Similarly, for students higher in attachment avoidance, therapy can help them first understand the deactivating strategies and specifically the suppression of their feelings. Therefore, students with attachment avoidance can find more healthy ways to come in touch with their emotions. By strengthening emotional regulation, they can realize that overreliance on the self is a maladaptive coping mechanism that is used to maximize distance from others and used for emotional cut-off, which is very unhelpful in the long-term as it is linked with depression and anxiety [78,79].

Moreover, except for assessing and working with students’ attachment dimensions, therapeutic interventions can focus directly on developing and strengthening resilience. Past studies suggested some interventions, which can facilitate resilience such as mindfulness [80], resilience journal intervention [81], and coping intervention, which is provided on a group level [82]. In addition, in a scoping review, it was reported that cognitive behavioral interventions, psychoeducation, and other interventions focusing on issues of self-efficacy and self-esteem could also be proven useful in enhancing university students’ resilience [83].

Finally, universities can implement some communication strategies to bring to students’ attention issues related to attachment insecurity, psychological resilience, and well-being. This can have a preventive function firstly by educating students on important aspects of their personal development and growth and secondly by making counseling services more accessible, specifically for those students who may be considered at risk. University management must work closely with their counseling services but also with student disability offices, faculty members, student unions, student affairs offices, and generally with staff involved in students’ academic experience. Lastly, if universities want to offer the best possible options in supporting their students and increasing their LS, then the most recent evidence-based research should be followed, with special attention on the post-pandemic effects on university students’ experience.

## Data Availability

The data of this study are unavailable due to ethical restrictions.

## Supplementary Materials

The following supporting information can be downloaded at: https://www.mdpi.com/article/10.3390/ijerph21010022/s1, Table S1: Internal Consistency for attachment dimensions, resilience and LF; Table S2: Demographic characteristics of the sample; Table S3: Descriptive statistics; Table S4: Spearman rho correlations coefficients between main variables; Table S5: Mediation analysis with attachment anxiety as the independent variable; Table S6: Mediation analysis with attachment avoidance as the independent variable; Figure S1: Model for mediation of psychological resilience in the relationship between attachment anxiety and LS; Figure S2: Model for mediation of psychological resilience in the relationship between attachment avoidance and LS.
