# Supplementary material for: Attachment, Resilience and Life Satisfaction of University Students in Cyprus after the Fourth Wave of COVID-19
Source: Int J Environ Res Public Health. 2023 Dec 22;21(1):22. doi: 10.3390/ijerph21010022 (PMC10815057; doi:10.3390/ijerph21010022)
Supplement: Supplementary file 1 [file ijerph-21-00022-s001.zip › ijerph-2718862-supplementary.pdf]

Table S1. Internal Consistency for attachment dimensions, resilience and LF.

Scales	Cronbach's Alpha
Attachment Anxiety	0.906
Attachment Avoidance	0.916
Total Resilience	0.907
Resilience Self-efficacy	0.872
Resilience Self-Confidence	0.850
Life Satisfaction	0.899

Table S2. Demographic characteristics of the sample.

Demographic Variable	University Students (*n* = 780)
Gender:	
Men	41.4% (*n* = 323)
Women	58.6% (*n* = 457)
Nationality:	
Cypriots	45.8% (*n* = 357)
Greeks	54.2% (*n* = 423)
Marital Status:	
Single	88.2% (*n* = 688)
Married/Engaged	10.3% (*n* = 80)
Divorced	1.3% (*n* = 10)
Widowed	0.3% (*n* = 2)
Residence Status:	
Living Alone	47.3% (*n* = 369)
Living with partner	13.8% (*n* = 108)
Living with Parents	27.7% (*n* = 216)
Living with Roommates	11.2% (*n* = 87)
Level of Study:	
Diploma	6.3% (*n* = 49)
Bachelor	78.3% (*n* = 611)
Master	14.6% (*n* = 114)
Doctorate	0.8% (*n* = 6)
Program of Study:	
Medicine	9.5% (*n* = 74)
Sciences	24.5% (*n* = 191)
Social Sciences	34.5% (*n* = 269)
Humanities	5.6% (*n* = 44)
Education	7.6% (*n* = 59)
Business/Economics/Management	15% (*n* = 117)
Other	3.% (*n* = 26)
Year of Study:	
First	20.5% (*n* = 160)
Second	27.4% (*n* = 214)
Third	19.6% (*n* = 153)
Fourth	24.9% (*n* = 194)
Fifth	6.9% (*n* = 54)
Sixth+	0.6% (*n* = 5)
Mean Age	22.00 (*SD* = 3.39)

Table S3. Descriptive statistics.

	Attachment Anxiety	Attachment Avoidance	Life Satisfaction	Resilience Self-Efficacy	Resilience Self-Confidence	Resilience Total
Mean	3.25	3.32	23.08	16.79	8.75	25.54
Median	3.22	3.39	23.00	17.00	9.00	26.00
Std. Deviation	1.13	1.12	7.00	4.95	2.80	7.22
Minimum	1.00	1.00	5.00	0.00	0.00	0.00
Maximum	6.67	6.89	35.00	24.00	12.00	36.00
IQR	1.67	1.72	10.00	7.00	4.00	10.00

Table S4. Spearman rho correlations coefficients between main variables.

	1	2	3	4	5	6
1. Attachment Anxiety	------					
2. Attachment Avoidance	.377**	------				
3. Life Satisfaction	-.371**	-.440**	------			
4. Resilience Self-Efficacy	-.264**	-.289**	.485**	------		
5. Resilience Self-Confidence	-.377**	-.360**	.591**	.699**	------	
6. Resilience Total	-.335**	-.341**	.563**	.957**	.868**	------

Note. Total n = 780, ** $p < 0.001$.

Table S5. Mediation analysis with attachment anxiety as the independent variable.

Predictor	b	95%CIs	t	Sig	F (df)	R^2	Sig
					Model 1		
Attachment Anxiety	-1.986	[-2.416 -1.557]	-9.08	p<0.001	82.5 (1, 778)	0.096	p<0.001
					Model 2		
Resilience	0.504	[0.447 0.560]	17.43	p<0.001			
Attachment Anxiety	-1.274	[-1.638 -0.910]	-6.87	p<0.001	235.14 (2, 777)	0.377	p<0.001
					Total Effect		
Total	-2.274	[-2.682 -1.866]	-10.95	p<0.001	119.86 (1, 778)	0.133	p<0.001
					Indirect Effect		
Attachment Anxiety	-1.000	[-1.265 -0.757]	-8.05*	p<0.001			

Note. * Based on Sobel statistic. Dependent variable: Life Satisfaction.

Table S6. Mediation analysis with attachment avoidance as the independent variable.

Predictor	b	95%CIs	t	Sig	F (df)	R^2	Sig
					Model 1		
Attachment Avoidance	-2.167	[-2.593 -1.741]	-9.99	p<0.001	99.7 (1, 778)	0.114	p<0.001
					Model 2		
Resilience	0.476	[0.420 0.532]	16.69	p<0.001			

Attachment Avoidance	-1.696	[-2.055 -1.336]	-9.25	p<0.001	264.10 (2, 777)	0.405	p<0.001
					Total Effect		
Total	-2.727	[-3.122 -2.333]	-13.56	p<0.001	183.95 (1, 778)	0.191	p<0.001
					Indirect Effect		
Attachment Avoidance	-1.032	[-1.310 -0.780]	-9.25*	p<0.001			

Note. * Based on Sobel statistic. Dependent variable: Life Satisfaction.

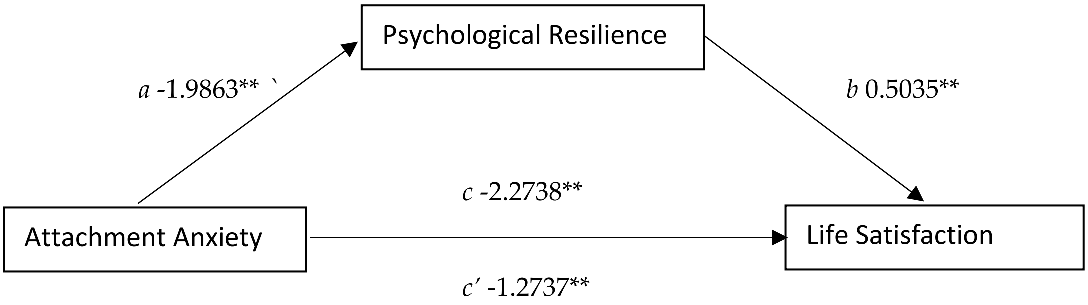

Figure S1. Model for mediation of psychological resilience in the relationship between attachment anxiety and LS.

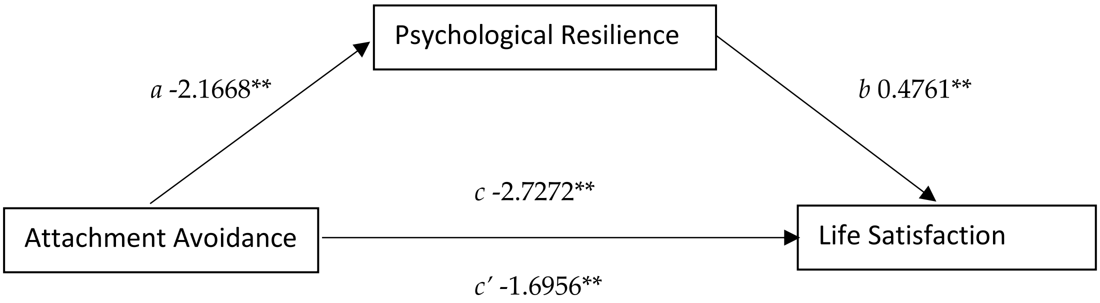

Figure S2. Model for mediation of psychological resilience in the relationship between attachment avoidance and LS.
